# The Effects of Salinity on Microbial Metabolic Limitation and Carbon Use Efficiency in Rhizosphere and Bulk Soils of *Tamarix austromongolica*

**DOI:** 10.3390/plants15030344

**Published:** 2026-01-23

**Authors:** Jia Sun, Jianmin Chu, Jingbo Wang, Qian Wang

**Affiliations:** 1 Research Institute of Forestry, Chinese Academy of Forestry, Beijing 100091, China; sunjia009900@163.com (J.S.);; 2 Experimental Center of Desert Forestry, Chinese Academy of Forestry, Bayannur 015200, China; 3 College of Forestry, Shenyang Agricultural University, Shenyang 110866, China

**Keywords:** salinity, rhizosphere, ecoenzymatic stoichiometry, microbial metabolic limitation, microbial carbon use efficiency

## Abstract

Soil extracellular enzyme activity reflects microbial resource acquisition and metabolic efficiency. However, applying enzyme stoichiometry to explore microbial metabolic limitations and carbon use efficiency (CUE) in rhizosphere and bulk soils under saline conditions remains limited. In this study, rhizosphere and bulk soils of *Tamarix austromongolica* were sampled along a salinity gradient in the Yellow River Delta to assess microbial metabolic limitation and CUE. Results showed that increasing salinity intensified microbial metabolic limitations and markedly reduced CUE, identifying salinity as the dominant factor constraining microbial efficiency. Rhizosphere soils consistently exhibited phosphorus limitation, whereas bulk soils shifted from balanced N–P limitation to pronounced N limitation with increasing salinity. Despite stronger microbial C limitation, CUE remained significantly higher in the rhizosphere than in the bulk soils, suggesting that continuous carbon inputs and enhanced enzyme activity partially mitigated salinity-induced stress. These findings highlight the complex interplay between salinity stress and rhizosphere effects in regulating microbial nutrient acquisition and carbon metabolism. Overall, this study demonstrates the utility of enzyme stoichiometry for evaluating microbial functional adaptation in saline habitats and provides insights that may contribute to the theoretical basis for vegetation restoration in saline-alkali ecosystems.

## 1. Introduction

Soil salinization stands as one of the primary drivers of land degradation, posing a severe threat to soil health. Globally, approximately 9.32 × 10^9^ ha of land are affected by varying degrees of salinization. Projections indicate that by 2050, 50% of the world’s arable land will experience drought or salinization [1]. The resulting high osmotic stress and ionic toxicity caused by soil salinization jeopardize the survival of soil microorganisms and impair their ecological functions [2].

Soil extracellular enzymes, secreted by microorganisms to acquire nutrients, regulate organic matter decomposition and nutrient (C, N, P) absorption [3,4], influencing ecosystem functionality and productivity [5]. Its activity ratio indicates the biogeochemical equilibrium between microbial nutrient demands and environmental nutrient availability, and is used to characterize the nature and extent of microbial resource utilization strategies and microbial metabolic constraints [6,7,8]. Nutrient limitation affects the metabolic rate of microorganisms and the retention of C in soil [9,10]. Therefore, microbial metabolic limitations are the key factor in controlling the C cycle of terrestrial ecosystems. Elucidating microbial metabolic limitation patterns and their underlying drivers is essential for understanding and regulating critical soil C processes.

Current studies have revealed the prevalence of microbial metabolic limitation [8,11,12] and are largely shaped by environmental factors [13,14,15,16]. However, most studies treat the soil as a homogeneous system or focus only on the rhizosphere environment, while ignoring the heterogeneous nature of the rhizosphere and bulk soils, which have a differential impact on the metabolic strategies of microorganisms. As the critical interface of plant–soil-microbe interactions, the rhizosphere exhibits more active material transformation and energy fluxes than bulk soil [17]. Root activities may reshape microbial resource allocation patterns through exudate inputs and nutrient competition. For instance, studies demonstrate stronger microbial C and N limitations in rhizosphere soil than in the bulk soil of *Pinus sylvestris* var. *Mongolica plantations* [18]. Similarly, enhanced microbial P limitation occurs in the rhizosphere of Pinus armandii under N deposition compared to bulk soil [19]. However, such rhizosphere-mediated metabolic differentiation remains inadequately explored in coastal wetland ecosystems, especially within fragile habitats characterized by high salinity and low organic C content. Microbial carbon use efficiency (CUE) is defined as the ratio of C allocated to microbial growth versus total C assimilated, reflecting the balance between C loss and accumulation [20]. Elevated CUE signifies efficient C sequestration in microbial biomass, reducing atmospheric C release and enhancing soil C stabilization, which is beneficial for the stability of soil C and the mitigation of climate change [15]. Studies demonstrate that microbial CUE is modulated by microbial metabolic limitation and nutrient accessibility [21], with nutritional constraints typically suppressing CUE [22,23]. He et al. utilized ecoenzymatic stoichiometry to discover that CUE in agricultural, forest, and grassland ecosystems is negatively governed by microbial C limitation [24], confirming that resource constraints regulate the resource allocation of microorganisms [25,26]. Nevertheless, the specific mechanisms and relative importance by which microbial C/N/P limitations regulate CUE across divergent ecological niches and pronounced environmental gradients remain unresolved. In salinized regions, elevated soil salinity and constrained nutrient availability depress net primary productivity and microbial substrate use efficiency, thereby impeding organic C accumulation. Previous studies identify soil salinity as a master regulator of soil C stocks [27], soil C decomposition [28], and microbial activities [29], and in salt-affected ecosystems. Consequently, elucidating soil microbial CUE, metabolic limitation patterns, and their drivers is essential for formulating effective management strategies in salinized ecosystems.

The Yellow River Delta, situated at the ecotone where atmosphere, riverine flows, marine influences, and terrestrial systems converge, exhibits unique ecosystem characteristics shaped by dynamic interactions. Salinization poses a particularly severe challenge in this region. *Tamarix austromongolica*, as a pioneer species in saline-alkaline soils, plays a critical role in improving soil microenvironments through its extensive root system and salt-tolerant traits. However, current understanding remains markedly deficient regarding microbial metabolic limitation patterns in its rhizosphere versus bulk soils. Moreover, the rhizosphere effect on microbial CUE in saline-alkaline habitats is yet to be elucidated. Based on the identified research gaps, we propose the following central hypothesis: Along a salinity-alkalinity gradient, the rhizosphere zone of *T. austromongolica* alleviates microbial metabolic limitations and significantly enhances microbial CUE, while this effect is not significant in bulk soil. Furthermore, the relationship between microbial metabolic limitation and CUE differs significantly between the rhizosphere and bulk soil regions.

To test the above hypothesis, this study selected 32 sampling sites of *T. austromongolica* in the coastal saline-alkali area of the Yellow River Delta, collecting both rhizosphere and bulk soil samples from each. By measuring the basic physical and chemical properties of the soil, microbial extracellular enzyme activities (β-1,4-glucosidase, β-1,4-N-acetylglucosaminidase, L-leucine aminopeptidase, and alkaline phosphatase) were used to estimate the degree of microbial metabolic limitation and microbial CUE. This study aims to advance mechanistic understanding of soil microbial metabolic limitations and key regulators of CUE in coastal saline-alkaline ecosystems, ultimately providing a theoretical framework for assessing soil microbial C sequestration potential in China’s coastal salt-affected lands.

## 2. Results

### 2.1. Soil Biotic and Abiotic Properties

As shown in Table 1, both rhizosphere and bulk soils were dominated by silt and sand fractions, which together accounted for more than 80%. With increasing salinity, soil nutrient contents (SOC, DOC, TN, NO_3_^−^-N, TP, AP) and microbial biomass (MBC, MBN, MBP) showed significant decreases, although values in rhizosphere soils remained consistently higher than those in bulk soils. In contrast, NH_4_^+^-N content exhibited a significant increase along the salinity gradient (*p* < 0.05). Under low- and medium-salinity levels, the soil C:N ratio in the rhizosphere was significantly lower than that in the bulk soil, while the soil N:P ratio was significantly higher. However, no significant differences were observed under high-salinity levels. Both the soil C:P and N:P ratios in the rhizosphere decreased significantly with increasing soil salinity. The MBC:MBN ratio in rhizosphere soil was 41.74% and 37.39% lower under medium- and high-salinity levels than under low-salinity levels.

### 2.2. Extracellular Enzyme Activities (EEAs) and Ecoenzymatic Stoichiometry

**Figure 1:**
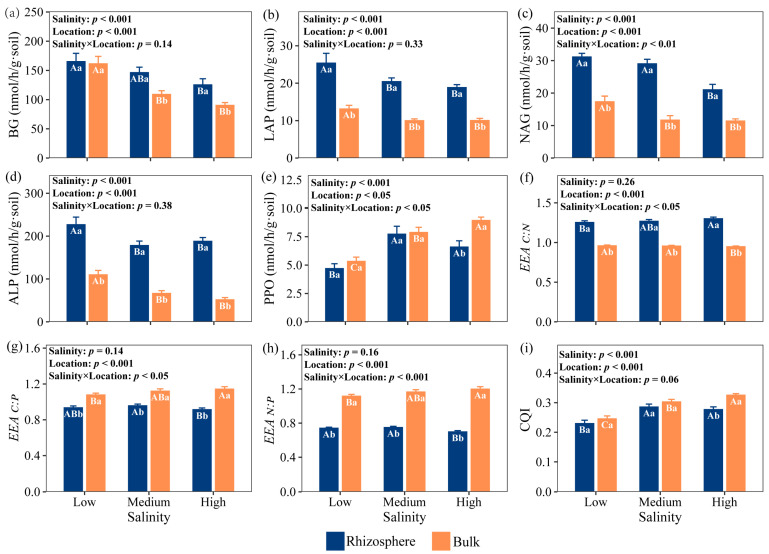
Extracellular enzyme activities and ecoenzymatic stoichiometry in rhizosphere and bulk soils under three salinity levels, (**a**) BG; (**b**) LAP; (**c**) NAG; (**d**) ALP; (**e**) PPO; (**f**) *EEA_C:N_*; (**g**) *EEA_C:P_*; (**h**) *EEA_N:P_*; (**i**) CQI. Different letters indicate significant differences at the 0.05 level. Uppercase letters indicate significant differences among the three salinity levels within the same soil location (rhizosphere or bulk), while lowercase letters indicate significant differences between rhizosphere and bulk soils under the same salinity level. Error bars represent standard error. Low salinity, *n* = 10; medium salinity, *n* = 12; high salinity, *n* = 10. BG, β-1,4-glucosidase; LAP, L-leucine aminopeptidase; NAG, β-1,4-N-acetylglucosaminidase; ALP, alkaline phosphatase. *EEA_C:N_* is the ratio of C and N-acquiring enzyme activities, lnBG/ln(NAG + LAP). *EEA_C:P_* is the ratio of enzyme activity lnBG/lnALP. *EEA_N:P_* is the ratio of enzyme activity ln(NAG + LAP)/lnALP. CQI is the ratio of enzyme activity lnPPO/ln(PPO + BG).

Salinity and location had significant main effects on the EEAs (*p* < 0.001), but their interactive effect was only significant on the N-acquiring enzyme activities (LAP and NAG) and PPO (*p* < 0.001; Figure 1). The EEAs generally decreased with the increase in salinity, but the PPO generally increased with the increase in salinity. Compared to low-salinity, the BG activity of the rhizosphere soil decreased by 11.36% and 23.78%, and that of the bulk soil decreased by 32.23% and 43.80% under medium- and high-salinity levels, respectively. Under the same salinity, the rhizosphere soil EEAs were greater than those in the bulk soil. Under low-, medium-, and high-salinity levels, the ALP activity in rhizosphere soil is 2.05 times, 2.66 times, and 3.60 times higher than that in bulk soil, respectively.

Salinity had no significant effect on the *EEA_C:N_*, *EEA_C:P_*, and *EEA_N:P_* (*p* > 0.05), but location had a highly significant effect on these (*p* < 0.001; Figure 1e–h). The *EEA_C:N_* in the rhizosphere was significantly higher than the bulk soil, whereas the *EEA_C:P_* and *EEA_N:P_* were significantly lower than the bulk soil (*p* < 0.05; Figure 1e–h). Furthermore, the CQI in the bulk soil under high-salinity levels was significantly higher than that under low- and medium-salinity levels. Under high-salinity levels, the CQI of bulk soil was significantly higher than that of rhizosphere soil, which was 17.86% higher than that of rhizosphere soil (*p* < 0.05; Figure 1i).

### 2.3. Soil Microbial Metabolic Limitation

**Figure 2:**
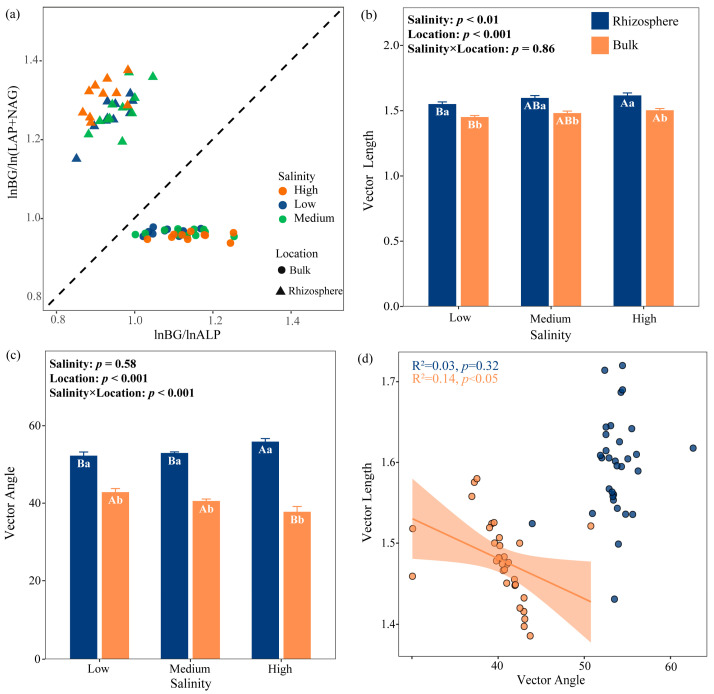
Relationships between ln(BG)/ln(NAG + LAP) versus ln(BG)/ln (ALP) at all sites (**a**), the variation in vector length and angle (**b**,**c**), and their relationships (**d**). Uppercase letters indicate significant differences among the three salinity levels within the same soil location (rhizosphere or bulk), while lowercase letters indicate significant differences between rhizosphere and bulk soils under the same salinity level. Error bars represent standard error. Low salinity, *n* = 10; medium salinity, *n* = 12; high salinity, *n* = 10. BG, β-1,4-glucosidase; LAP, L-leucine aminopeptidase; NAG, β-1,4-N-acetylglucosaminidase; ALP, alkaline phosphatase. Vector length represents soil C limitation for microbes. The vector angle represents the soil N/P limitation for microbes. A vector angle < 45° indicates N limitation, and a vector angle > 45° indicates P limitation.

Salinity and location had significant main effects on the vector length (microbial C limitation) (*p* < 0.05; Figure 2b). Vector analysis revealed significantly stronger microbial C limitation with increasing salinity, and the microbial C limitation in rhizosphere soil was significantly higher than in bulk soil (Figure 2b). In relation to bulk soil, the vector length increased by 6.85%, 8.11%, and 8.00% in rhizosphere soil under low-, medium-, and high-salinity, respectively. Salinity, location, and their interaction had a highly significant effect on the vector angles (*p* < 0.05; Figure 2c). In rhizosphere soil, microbial activity exhibits P limitation. Whereas in bulk vector angle consistently remains below 45°, approaching 45° under low- and medium-salinity levels, which indicates a potential metabolic balance between N and P limitation, and vector angle significantly decreased while N limitation was enhanced under high-salinity levels. And both N and P limitations intensify with elevated salinity levels (Figure 2c). Vector length and vector angle correlated negatively in rhizosphere soil (*p* < 0.05), but not in bulk soil (Figure 2d).

### 2.4. Soil Microbial Carbon Use Efficiency

**Figure 3:**
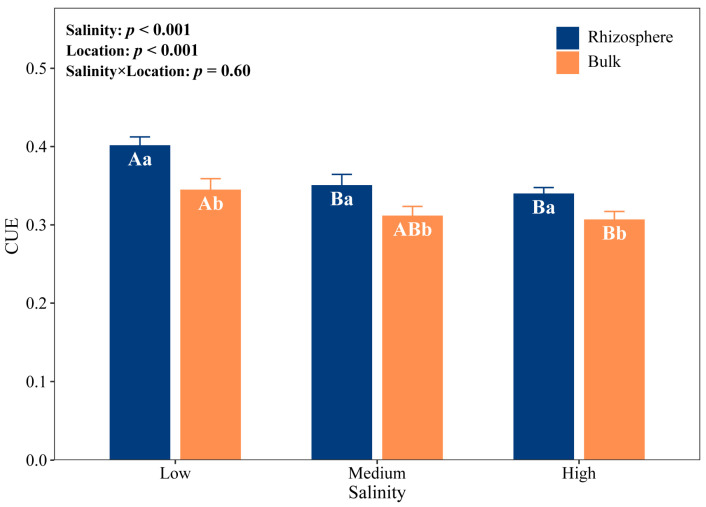
The microbial carbon use efficiency (CUE) in rhizosphere and bulk soils under three salinity levels. Uppercase letters indicate significant differences among the three salinity levels within the same soil location (rhizosphere or bulk), while lowercase letters indicate significant differences between rhizosphere and bulk soils under the same salinity level. Error bars represent standard error. Low salinity, *n* = 10; medium salinity, *n* = 12; high salinity, *n* = 10.

Figure 3 showed that salinity and location had significant main effects on the microbial CUE (*p* < 0.05). The microbial CUE of rhizosphere and bulk soil decreased with the increase in salinity. The microbial CUE of rhizosphere soil under medium- and high-salinity levels was significantly lower than that under low-salinity levels, which were 12.50% and 15.00% lower than that under low-salinity levels, respectively. CUE was significantly higher in the rhizosphere than in the bulk soil. In relation to bulk soil, the microbial CUE increased by 17.65%, 12.90%, and 16.67% in rhizosphere soil under low-, medium-, and high-salinity, respectively.

### 2.5. Relationship Between Soil Abiotic-Biotic Factors and Microbial Metabolic Limitation and CUE

**Figure 4:**
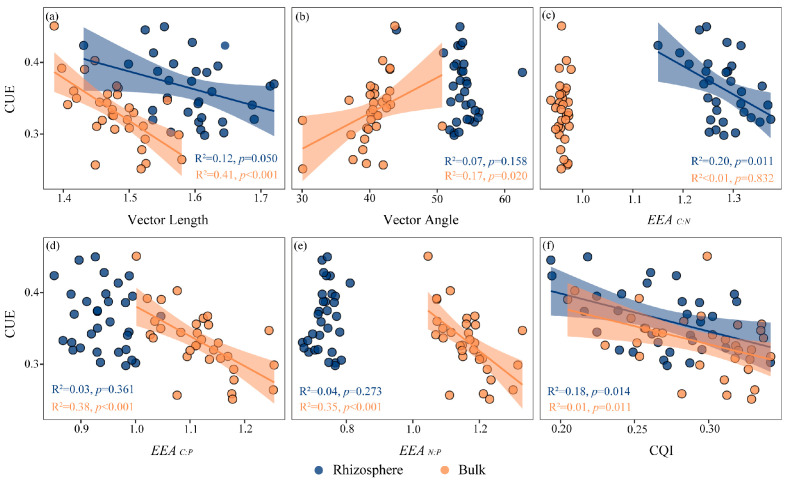
Linear relationships between microbial CUE and microbial metabolic limitation (**a**,**b**), and ecoenzymatic stoichiometry (**c**–**f**). *EEA_C:N_* is the ratio of C and N-acquiring enzyme activities, lnBG/ln(NAG + LAP). *EEA_C:P_* is the ratio of enzyme activity lnBG/lnALP. *EEA_N:P_* is the ratio of enzyme activity ln(NAG + LAP)/lnALP. CQI is the ratio of enzyme activity lnPPO/ln(PPO + BG).

**Figure 5:**
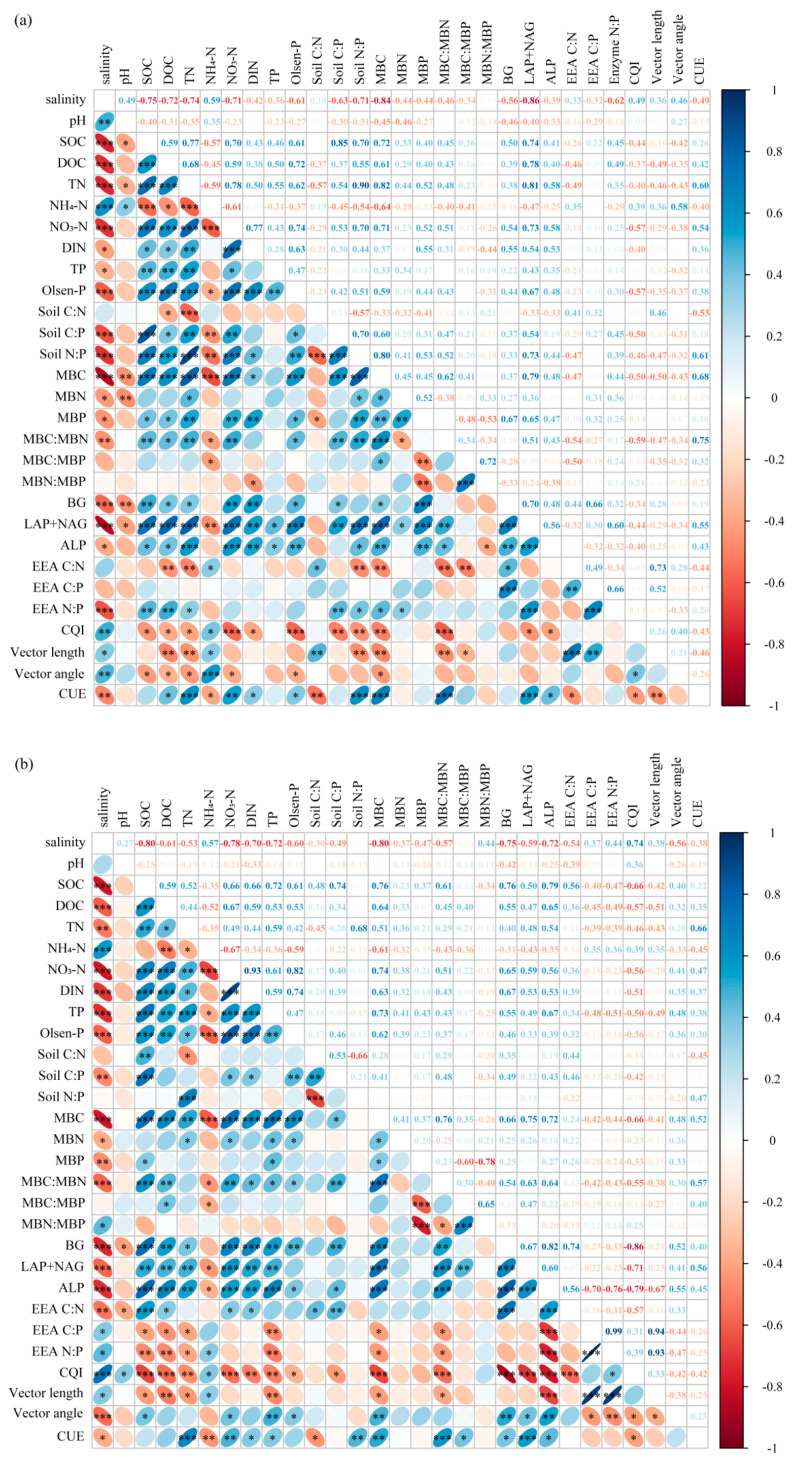
Correlation heatmaps based on Pearson correlation analysis in rhizosphere (**a**) and bulk soils (**b**). *** *p* < 0.001, ** *p* < 0.01, * *p* < 0.05. DIN, NH_4_^+^-N + NO_3_^−^-N; BG, β-1,4-glucosidase; LAP, L-leucine aminopeptidase; NAG, β-1,4-N-acetylglucosaminidase; ALP, alkaline phosphatase. Vector length represents soil C limitation for microbes. EEA C:N is the ratio of C and N-acquiring enzyme activities lnBG/ln(NAG + LAP). EEA C:P is the ratio of enzyme activity lnBG/lnALP. EEA N:P is the ratio of enzyme activity ln(NAG + LAP)/lnALP. CQI is the ratio of enzyme activity lnPPO/ln(PPO + BG). The size of the bubbles indicates the magnitude of the absolute value of the Pearson r value. The larger the bubble, the smaller the Pearson r value.

**Figure 6:**
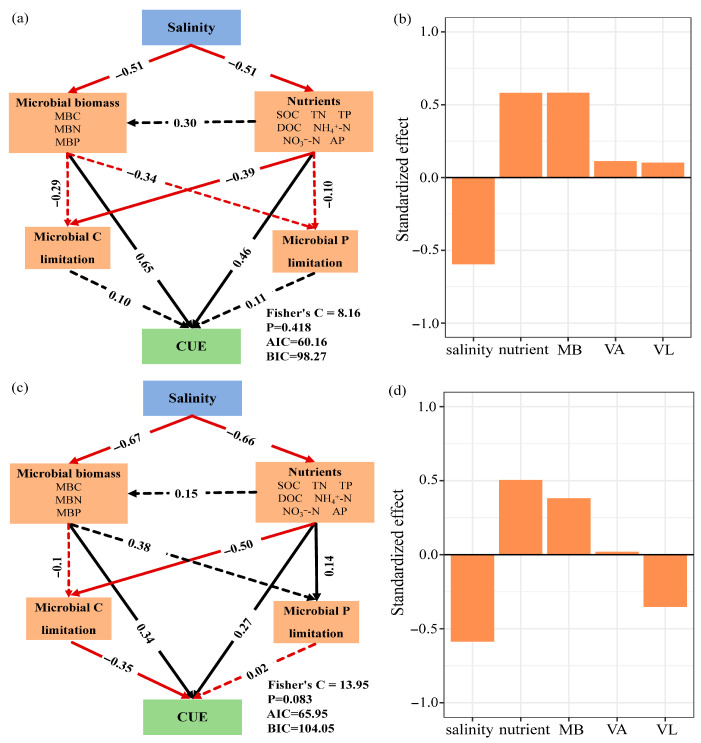
Pathway relationships depicting the effects of salinity, microbial biomass, nutrients, microbial C limitation, and microbial N/P limitation on microbial CUE were analyzed using structural equation modeling in rhizosphere (**a**) and bulk soil (**c**). Standardized total effects in rhizosphere (**b**) and bulk soil (**d**). Black and red arrows show positive and negative relationships, respectively. The real arrows and the dotted arrows represent the significant and insignificant relationships, respectively, and the numbers adjacent to the arrows represent standardized path coefficients. MB, microbial biomass; VA, microbial N/P limitation; VL, microbial C limitation.

Figure 4 showed that CUE had a significant negative correlation with VL (*p* = 0.05), *EEA_C:N_* (*p* < 0.05), and CQI (*p* < 0.05) in the rhizosphere soil. The CUE had a significant negative correlation with VL (*p* < 0.001), *EEA_C:P_* (*p* < 0.001), *EEA_N:P_* (*p* < 0.001), and CQI (*p* < 0.05), and had a significant positive correlation with VA (*p* < 0.05) in the bulk soil. The Pearson correlation between each of the indicators was analyzed (Figure 5). Piecewise-SEM indicated that salinity, nutrients, microbial biomass, microbial C limitation, and N/P limitation had direct or indirect effects on CUE (Figure 6). In the rhizosphere and bulk soil, the salinity had the highest total effects on CUE, and nutrients had strong positive effects on CUE (Figure 6a,b). In the rhizosphere soil, changes in the nutrients and microbial biomass induced by salinity significantly affected CUE (Figure 6a). In the bulk soil, salinity changed microbial C limitation by affecting nutrients, and ultimately affected CUE (Figure 6b).

## 3. Discussion

### 3.1. Microbial C Limitation

This study reveals pervasive microbial C limitation in soils of *T. austromongolica* across the Yellow River Delta’s salinized regions, with intensifying constraints under elevated salinity (Figure 2). First, salinization affects the content of effective substrate [27], which is the main energy and C source of microorganisms. Salinity restriction mainly affects plant growth and primary productivity of vegetation by changing soil osmotic potential and matrix potential, and affects the input, transformation, and spatial distribution pattern of soil organic matter and available nutrients [30]. Meanwhile, the increase in soil salinity will reduce the distribution of root biomass of *T.chinensis* to reduce the damage of salt to roots [31], resulting in a decrease in rhizosphere C deposition. This is corroborated by declining SOC and DOC with increasing salinity in our data (Table 1 and Figure 5). Furthermore, salinity-induced osmotic stress destabilizes enzyme structures, suppressing C-acquiring enzyme activities [32], as evidenced by reduced C-degrading enzyme function at higher salinity levels (Figure 1), rendering limited organic C less bioavailable. Critically, microbes expend substantial ATP to synthesize osmolytes for ion homeostasis under high Na^+^ and Cl^−^ stress, exacerbating C demands. Notably, Zhai et al. observed reduced microbial C limitation along a salinity gradient from freshwater (0.1‰) to low-salinity (2.1‰) in tidal wetlands—a range corresponding to our low-salinity treatments [33]. This divergence suggests a threshold effect in salinity responses, where metabolic constraints transition from moderate to severe beyond critical ionic concentrations.

Plant roots secrete substantial labile organic C compounds—including organic acids, sugars, and amino acids (collectively termed rhizodeposition)—into the rhizosphere, providing a rich C source for microorganisms. Consequently, the rhizosphere typically alleviates microbial C limitation relative to bulk soil [34]. However, stronger microbial C limitation was observed in rhizosphere soil across salinity gradients (Figure 2b), despite significantly higher DOC contents compared to bulk soil (Table 1). This is consistent with previous studies comparing rhizosphere and bulk soils [35,36]. This suggests that the enhanced C limitation observed in the rhizosphere does not stem from a shortage of available C substrates, but rather from an increase in microbial metabolic demand. The rhizosphere is a biologically active microzone enriched with root exudates that stimulate microbial growth, enzyme production, and the rapid turnover of labile organic matter. Such enhanced metabolic activity accelerates C cycling and quickly depletes the easily available C pools, creating a transient imbalance between C supply and demand [37,38]. In addition, the rhizosphere has a highly diverse and metabolically active microbial community, characterized by elevated population density, rapid growth, and strong enzymatic activity [39]. Such intensified microbial activity leads to fierce interspecific competition within the rhizosphere, as microorganisms compete for limited labile C and nutrients to sustain their high metabolic rates. At the same time, plant roots actively compete with microorganisms for nutrients and water, further constraining microbial access to soil resources and forcing them to invest additional C and energy into nutrient acquisition [40,41].

Moreover, a stronger P limitation was observed in the rhizosphere, as indicated by the larger vector angle (Figure 2c). To acquire sufficient phosphorus, microorganisms need to synthesize more phosphatase, which requires additional C and energy. This increased C investment, together with the intensified P limitation, may lead to a vicious cycle of ‘C-P co-limitation’. The simultaneously elevated activities of the C-acquisition enzyme BG and the P-acquisition enzyme ALP in the rhizosphere (Figure 1a,d) provide further evidence supporting this co-limitation pattern.

### 3.2. Microbial N/P Limitation

Elevated soil salinity intensifies microbial P limitation in the rhizosphere. On the one hand, the increase in salinity reduced the content of TP and AP (Table 1), and the ratio of salt to AP and inorganic N was significantly positively correlated (Figure 5). On the other hand, the synthesis of more structural molecules (e.g., DNA, RNA) in response to high salt osmotic stress leads to an increase in the demand for P [42], which intensifies the competition between plants and microorganisms. Concurrently, rising pH accompanying increased salinity (Figure 5) further diminishes phosphorus availability: elevated pH stimulates soil microbes to convert bioavailable P into immobilized organic forms through microbial fixation [43], while phosphate ions readily precipitate with free calcium ions, forming insoluble calcium phosphate [44]. Notably, Kang et al. observed weaker rhizosphere P limitation in oak forests during secondary succession, which was contrary to our results [35]. This may be due to the selective absorption and differential transformation of phosphorus by species.

Across salinity gradients, rhizosphere soil exhibits microbial P limitation, whereas bulk soil displays N limitation. Rhizosphere soil demonstrates higher *EEA_C:P_* and *EEA_N:P_* ratios than bulk soil (Figure 1g,h), with *EEA_N:P_* < 1 (Figure 1h), indicating more acute microbial P demand relative to C and N, thus confirming P limitation. This pattern is substantiated by vector analysis (Figure 2). Although the AP in the rhizosphere was higher than that in the bulk soil (Table 1), elevated microbial activity and diversity intensify intra-community competition for P, resulting in P limitation. When microorganisms are limited by P, plants and microorganisms compete fiercely for phosphorus [40]. Furthermore, *Tamarix* cultivation promotes conversion of labile P (Ca_2_-P) and moderately available P (Ca_8_-P, and Al-P) into recalcitrant P (Ca_10_-P), reducing soil P availability [45].

In bulk soil, vector angle consistently remains below 45°, approaching 45° under low- and medium-salinity levels, which may indicate a potential metabolic balance between N limitation and P limitation [24]. Lower microbial diversity and activity in bulk soil reduce nutrient demands, while *T. chinensis* shrubs enhance N and P enrichment (enrichment factors > 1) under low- and medium-salinity levels, which alleviates the limitation of N and P [46]. Under high-salinity levels, significantly reduced vector angle intensifies microbial N limitation due to salt-driven suppression of key N-transformation processes. The high salt environment inhibited the activity of ammonia monooxygenase (AMO), reduced the oxidation of NH_4_^+^ to NO_2_^−^, and then reduced the production of NO_3_^−^. Salt stimulated the expression of denitrification genes (e.g., nirKnirK and nirSnirS) by increasing osmotic pressure, and promoted the reduction of NO_3_^−^ to N_2_O, which led to the reduction of NO_3_^−^-N supply [26]. The significant negative correlation between salinity and NO_3_^−^-N observed in our study further substantiates this mechanism (Figure 5). The ion toxicity caused by high salt stress reduced the activity of LAP and NAG (Figure 1 and Figure 5), and the ability of microorganisms to obtain nitrogen also decreased. Yang et al. investigated *Larix principis-rupprechtii* plantations in North China and found that both rhizosphere and bulk soils exhibited microbial N limitation [47], suggesting that differences among tree species may also have a substantial influence on microbial metabolic limitations.

### 3.3. Microbial Carbon Use Efficiency

Elevated salinity significantly reduced microbial CUE in both rhizosphere and bulk soils (Figure 3), and salinity was the dominant driver of CUE decline (Figure 6b,d). Salinity primarily affects microbial CUE through its influence on microbial biomass and nutrient availability (Figure 6a,c). SOC and DOC were significantly positively correlated with CUE (Figure 5), while the increase in salinity reduced SOC and DOC (Table 1), and the depletion of bioavailable C substrates directly suppresses CUE. To adapt to salt stress, microorganisms optimize genomic configurations to exploit ecological niches [2]. Maintaining osmotic pressure by activating ion pump systems and accumulating organic solutes [48,49], these processes require a large amount of C and energy input, which in turn leads to the redistribution of intracellular C and reduces the C resources that should be used for microbial growth [50]. The negative correlation between microbial biomass and salinity also proved this (Figure 5 and Figure 6).

The strong negative correlation between microbial C limitation and CUE in both rhizosphere and bulk soils aligns with prior research demonstrating that resource limitation regulates resource-acquisition strategies (Figure 4a) [24]. Interestingly, microbial CUE was consistently and significantly elevated in the rhizosphere compared to bulk soil across all salinity levels, despite a concurrent intensification of C limitation (Figure 2 and Figure 3). We attempted to explain this paradoxical phenomenon through CQI but found that the difference in CQI between rhizosphere and bulk soils was only significant under high-salinity levels (Figure 1g). Moreover, although the increase in ALP activity in the rhizosphere entails substantial energy and C costs, it did not lead to a decline in CUE. This observation further supports the existence of a multifaceted, rhizosphere-specific strategy for maintaining high CUE.

First and fundamentally, the continuous input of highly labile primary metabolites (root exudates) provides a direct, readily assimilable C subsidy that minimizes the metabolic cost of enzyme synthesis for microbial growth [51,52]. This mechanism operates consistently across all salinity levels. At the same time, the rhizosphere benefits from this steady influx of labile C, which helps to offset the substantial energetic costs associated with enhanced ALP synthesis under strong P limitation. Therefore, the previously described ‘P-C co-limitation’ primarily reflects potential resource competition, while its negative impact on CUE is likely mitigated by these compensatory C inputs.

Second, the elevated enzyme activity in the rhizosphere (Figure 1a–c) originates not only from microbes but also from root-derived enzymes [53]. Critically, root-derived enzymes can initiate the decomposition and depolymerization of complex organic compounds (e.g., polysaccharides and lignin derivatives), effectively ‘pre-processing’ these substrates and increasing the pool of bioavailable C available to microorganisms [54]. This exogenous catalytic activity may reduce the metabolic investment required by microbes for enzyme production, thereby lowering their initial C and energy costs for nutrient acquisition. This process may indirectly promote microbial CUE by alleviating substrate limitation, although further investigation is needed to confirm this mechanism.

Finally, the significantly higher dissolved inorganic nitrogen (DIN) in the rhizosphere (as indicated by the significant positive correlation between CUE and DIN in Figure 5) crucially supports microbial growth. Enhanced nitrogen availability allows microorganisms to efficiently incorporate C into biomass, thereby increasing CUE [55]. This is consistent with the positive correlation between CUE and VA in bulk soil (Figure 4b), where enhanced nitrogen limitation conversely reduces CUE.

In summary, the consistently higher CUE in the rhizosphere is driven by a synergistic combination of continuous labile C input, supplemental enzyme activity, and improved nitrogen availability. The observed shift to a lower CQI at high salinity in the rhizosphere (Figure 1g) then acts as a reinforcing mechanism under stress, further enriching the pool of bioavailable C and helping to offset the heightened osmotic costs, thereby robustly bolstering soil C sequestration across the salinity gradient.

## 4. Materials and Methods

### 4.1. Study Area

Our study area is located in the Yellow River Delta (37°20′–38°12′ N, 118°07′–119°10′ E). Influenced by sea–land interactions, the region forms a geomorphic pattern ranging from plain to tidal flat from Southwest to Northeast, with an overall flat topography. The Yellow River Delta has a mean annual temperature of 11.7–12.6 °C and a mean annual precipitation of approximately 530–630 mm, about 75% of which occurs from June to September. Evaporation substantially exceeds precipitation, resulting in a pronounced seasonal water imbalance. According to the Köppen–Geiger climate classification, the area is predominantly categorized as Cwa (temperate monsoon climate with dry winters and wet summers) [56]. Large-scale soil reclamation in the Yellow River Delta began in 1956, marking the onset of human-induced land conversion for agriculture and settlement. Subsequent reclamation activities further accelerated the transformation of coastal alluvial soils into coastal saline soils, accompanied by vegetation destruction and wetland degradation. The soil type in this region is classified as salinized fluvo-aquic soil in the Chinese Soil Taxonomy, which corresponds to Solonchaks in the World Reference Base for Soil Resources [57]. The dominant plant species are *Robinia pseudoacacia*, *Fraxinus chinensis*, *T. austromongolica*, *Phragmites australis*, *Suaeda salsa*, *Imperata cylindrical*, *Aeluropus littoralis*, *Cynanchum chinense*, and other salt-tolerant plants.

### 4.2. Field Sampling

In July 2024, a total of 32 sampling points of *T. austromongolica* with relatively consistent environments were selected in the Yellow River Delta region. The sampling sites are shown in Table 2. A 30 m × 30 m square was set up for each sample point, and three 5 m × 5 m small quadrats were set up along a diagonal of the square as a repeat of the sample point. The basal diameter, plant height, and crown width of each *T. austromongolica* in the sample were measured, and one standard wood was selected in each small quadrat according to the above indicators. The roots of *T. austromongolica* were collected by the digging method, and the soil and roots (traced back to the trunk to ensure accurate identification of roots) were dug around the trunk of each target tree using tools such as spades, tweezers, and brushes. We collected the 2 mm thick soil attached to the surface of the fine roots, which was treated as rhizosphere soil, and the remaining soil (approximately 20 cm from the roots) was bulk soil. Since the roots of *T. austromongolica* are mainly distributed in the 0–50 cm soil layer [58], we collected the rhizosphere and bulk soils of 0–50 cm. Three replicates of each sample were mixed into one soil sample (i.e., one rhizosphere soil sample and one bulk soil sample per site). Soil subsamples were stored at −20 °C for measuring biotic properties and air-dried for analyzing abiotic properties.

### 4.3. Measurement of Soil Biotic and Abiotic Properties

Soil pH was measured using a pH electrode, with a water-to-soil ratio of 5:1. The soluble salt content was measured by the gravimetric method with a water-to-soil ratio of 5:1. To eliminate the interference of chloride ions on soil organic carbon (SOC) determination in salinized soils, a sulfuric acid pre-heating method was applied [59], followed by SOC content measurement using the potassium dichromate oxidation method [60]. Dissolved organic carbon (DOC) was extracted with 0.5 M K_2_SO_4_, and quantified using a total organic carbon (TOC) analyzer (TOC-VCPH, Shimadzu, Kyoto, Japan) [61]. Total N (TN) content was measured by the Kjeldahl method with a Kjeltec 8400 analyzer (FOSS Analytical, Hillerød, Denmark) [62]. Ammonium (NH_4_^+^) and nitrate (NO_3_^−^) were extracted using a 0.5 mol L^−1^ KCl solution, and analyzed using a continuous flow analyzer (AA3, Bran + Luebbe, Norderstedt, Germany) to determine the soil ammonium nitrogen (NH_4_^+^-N) and nitrate nitrogen (NO_3_^−^-N) contents. Total phosphorus (TP) and available phosphorus (AP) were extracted with H_2_SO_4_-HClO_4_ and sodium bicarbonate [63].

Soil microbial biomass carbon (MBC), nitrogen (MBN), and phosphorus (MBP) were determined using the chloroform fumigation–extraction method [64,65]. Fresh soil samples (equivalent to 5 g oven-dry weight) were divided into two subsamples. One subsample was fumigated with ethanol-free chloroform in a desiccator for 24 h at 25 °C in the dark to lyse microbial cells, while the other was left unfumigated as a control. After fumigation, the chloroform was removed by repeated evacuation, and both fumigated and unfumigated soils were extracted with 0.5 mol L^−1^ K_2_SO_4_ for MBC and MBN determination, and with 0.5 mol L^−1^ NaHCO_3_ for MBP determination. Extracts were filtered through filter paper. The organic C and total N in the K_2_SO_4_ extracts were analyzed using a TOC/TN analyzer, and total P in the NaHCO_3_ extracts was measured colorimetrically using the molybdate–ascorbic acid method. MBC, MBN, and MBP were calculated as the differences between fumigated and unfumigated samples divided by the corresponding conversion coefficients. The conversion coefficients for MBC, MBN, and MBP used in this study were 0.45, 0.54, and 0.40, respectively.

### 4.4. Determination and Calculation of Extracellular Enzyme Activities (EEAs) and Stoichiometry

The activities of one extracellular C-acquiring enzyme (β-1,4-glucosidase, BG), two N-acquiring enzymes (β-1,4-N-acetylglucosaminidase, (NAG) and L-leucine aminopeptidase (LAP)), one P-acquiring enzyme (alkaline phosphatase, ALP), and one oxidase (Polyphenol oxidase, PPO) were assayed using standard fluorometric techniques. Specifically, 1 g of fresh soil was homogenized with 125 mL of 50 mM acetate buffer to create a soil suspension. Then, 200 μL of the suspension was aliquoted into a microplate to serve as the basis for the blank, standard, and substrate control wells. Subsequently, 50 μL of acetate buffer was added to the blank wells, 50 μL of the appropriate standard [4-methylumbelliferone (MUB) for BG, NAG, and ALP; 7-amino-4-methylcoumarin (AMC) for LAP; L-3,4-dihydroxyphenylalanine (L-DOPA) for PPO] was added to the standard wells, and 50 μL of the corresponding enzyme-specific substrate (4-MUB-β-D-glucoside for BG, 4-MUB-N-acetyl-β-D-glucosaminide for NAG, L-leucine-AMC for LAP, 4-MUB phosphate for ALP, and L-DOPA for PPO) was added to the substrate control wells. The hydrolysis reactions for the hydrolases were conducted by incubating the plate in the dark at 25 °C for 4 h, while the oxidation reaction for PPO was incubated for 5 h, after which all reactions were terminated with 10 μL of 1.0 M NaOH. Fluorescence intensity was measured using a multimode microplate reader (DTX 880 Multimode Detector, Beckman Coulter Inc., Fullerton, CA, USA) at excitation/emission wavelengths of 365/450 nm. Enzyme activities were finally expressed as nmol of substrate converted per hour per gram of dry soil (nmol h^−1^ g^−1^) [66].

According to Sinsabaugh et al. and Cui et al., *EEA_C:N_*, *EEA_C:P_*, and *EEA_N:P_* are the activity ratios among the C-acquiring enzyme, N-acquiring enzyme, and P-acquiring enzyme as Equations (1)–(3) [7,67].

(1) $${EEA}_{C:N}=lnBG/ln(LAP+NAG)$$

(2) $${EEA}_{C:P}=lnBG/lnALP$$

(3) $${EEA}_{N:P}=lnBG/lnALP$$

the C quality index (CQI) represents the fraction of refractory organic matter in enzyme-catalytic organic matter [68] and is calculated as Equation (4).

(4) CQI=lnPPO/(lnPPO+lnBG)

microbial metabolic limitation was assessed through vector models [69] and calculated via Equations (5) and (6):

(5) $$Vector\;length=\sqrt{{\left(lnBG/ln(NAG+LAP)\right)}^{2}+{\left(lnBG/lnALP\right)}^{2}}$$

(6) $$Vector\;angle=Degrees\left(ATAN2(\frac{lnBG}{lnALP}),(lnBG/ln(NAG+LAP))\right)$$

microbial C limitation increases with the vector length (VL). Vector angles (VA) > 45° indicate microbial P limitation, while VA < 45° indicates microbial N limitation. Microbial P limitation increased with VA, whereas N limitation decreased with increasing VA.

### 4.5. Calculation of Microbial Carbon Use Efficiency

Following the stoichiometric theory approach described by [24,70], microbial CUE was calculated based on the balance between elements required for microbial growth and elements available in the soil:

(7) $$CUE={CUE}_{max}\times {\left\{(S_{C:N}\times S_{C:P})/\left[\left(K_{C:N}+S_{C:N})\times (K_{C:P}+S_{C:P}\right)\right]\right\}}^{0.5}$$

(8) $$S_{C:N}=(1/{EEA}_{C:N})\times ({MB}_{C:N}/L_{C:N})$$

(9) $$S_{C:P}=(1/{EEA}_{C:P})\times ({MB}_{C:N}/L_{C:P})$$

where CUE_max_ represents the maximum microbial growth efficiency and is fixed to 0.6. K_C:N_ and K_C:P_ are half-saturation constants (0.5). EEA_C:N_, BG/(NAG + LAP); EEA_C:P_, BG/AP; MB_C:N_, MBC/MBN; MB_C:P_, MBC/MBP; L_C:N_, SOC/TN; L_C:P_, SOC/TP.

### 4.6. Data Analysis

We used the ‘quantile’ cluster analysis method to divide the 32 samples into three salinity levels. Specifically, soil salinity values were ordered and grouped based on empirical quantiles, resulting in three data-driven salinity classes: low (0.25–3.75 g·kg^−1^), medium (3.75–6.5 g·kg^−1^), and high (6.5–13 g·kg^−1^). The normality test and equal variance test were carried out for the original data to ensure the validity of subsequent parametric tests. Two-way analysis of variance (ANOVA) was applied to evaluate the main and interactive effects of salinity and location (rhizosphere and bulk soil) on soil chemical properties, extracellular enzyme activities (EEAs), ecoenzymatic stoichiometry, microbial metabolic limitation, and carbon use efficiency (CUE). Multiple comparisons were conducted using the least significant difference (LSD) test to identify significant group differences. Linear regression analysis was used to analyze the correlations between EEAs, ecoenzymatic stoichiometry, microbial metabolic limitation, and CUE. Finally, piecewise structural equation modeling (SEM) was used to assess the direct and indirect effects of environmental variables on microbial CUE. All statistical analyses were performed using R version 4.1.3. SEM analyses were conducted with the ‘piecewiseSEM,’ ‘nlme,’ and ‘lme4’ packages.

## 5. Conclusions

According to the extracellular enzyme stoichiometry, we infer pervasive microbial relative C limitation in both rhizosphere and bulk soils of *T. austromongolica*. In rhizosphere soil, microbial activity exhibits P limitation. Whereas in bulk vector angle consistently remains below 45°, approaching 45° under low- and medium-salinity levels, which indicates a potential metabolic balance between N limitation and P limitation, while N limitation was enhanced under high-salinity levels. Microbial C limitation was stronger in the rhizosphere than in the bulk soil, reflecting plant-microbe nutrient competition. Salinity increased microbial metabolic limitation in both soils and reduced microbial CUE. It is worth noting that although the rhizosphere C limitation was stronger, its CUE was significantly improved due to the ‘carbon-efficient strategy’. Soil salinity regulates the important factors of microbial community in salinized habitats and plays a major role in the change in CUE. Soil nutrients are the main drivers of CUE changes. These results highlight root effects on microbial metabolism and CUE, offering insights for nutrient management in salinized habitats.

## Figures and Tables

**Table 1:** Physical and chemical properties of rhizosphere and bulk soils under three salinity levels.

Indicator	Rhizosphere Soil			Bulk Soil		
	Low Salinity *n* = 10	Medium Salinity *n* = 12	High Salinity *n* = 10	Low Salinity *n* = 10	Medium Salinity *n* = 12	High Salinity *n* = 10
Clay (%)	12.10 ± 0.90 ABb	14.06 ± 0.78 Ab	11.64 ± 0.71 Bb	15.08 ± 0.56 Aa	15.93 ± 1.14 Aa	14.02 ± 0.74 Aa
Silt (%)	60.38 ± 2.15 Aa	59.74 ± 2.01 Aa	57.46 ± 1.85 Aa	59.08 ± 2.31 Aa	57.50 ± 2.15 Aa	61.90 ± 2.06 Aa
Sand (%)	27.52 ± 2.11 Aa	26.20 ± 1.95 Aa	30.90 ± 1.78 Aa	25.84 ± 2.26 Aa	29.57 ± 2.43 Aa	24.08 ± 12.17 Ab
Moisture content (%)	18.09 ± 1.05 Aa	18.02 ± 0.99 Aa	20.41 ± 1.51 Aa	20.19 ± 1.22 Aa	19.84 ± 1.41 Aa	19.65 ± 1.21 Aa
Salt content (g kg^−1^)	2.12 ± 0.27 Ca	4.57 ± 0.28 Ba	8.95 ± 0.56 Aa	2.24 ± 0.35 Ca	5.10 ± 0.28 Ba	9.63 ± 0.54 Aa
pH	8.30 ± 0.08 Ba	8.39 ± 0.06 Ba	8.70 ± 0.13 Aa	8.46 ± 0.11 Aa	8.54 ± 0.07 Aa	8.67 ± 0.12 Aa
SOC (g kg^−1^)	7.99 ± 0.73 Aa	5.76 ± 0.32 Ba	3.83 ± 0.37 Ca	7.32 ± 0.60 Aa	5.23 ± 0.38 Ba	3.79 ± 0.38 Ca
TN (g kg^−1^)	0.89 ± 0.07 Aa	0.61 ± 0.06 Ba	0.36 ± 0.05 Ca	0.39 ± 0.04 Ab	0.31 ± 0.03 ABb	0.24 ± 0.02 Bb
TP (mg kg^−1^)	596.48 ± 37.89 Aa	595.11 ± 19.88 Aa	462.07 ± 23.99 Aa	526.71 ± 26.55 Aa	462.07 ± 23.99 Ab	346.73 ± 18.89 Bb
DOC (mg kg^−1^)	229.56 ± 14.02 Aa	195.43 ± 15.04 Aa	134.64 ± 6.08 Ba	154.36 ± 10.40 Ab	127.67 ± 5.84 Bb	111.25 ± 6.98 Bb
NH_4_^+^-N (mg kg^−1^)	2.28 ± 0.22 Ca	3.73 ± 0.22 Ba	4.40 ± 0.23 Aa	1.77 ± 0.11 Ba	2.03 ± 0.16 Bb	2.57 ± 0.17 Ab
NO_3_^−^-N (mg kg^−1^)	5.45 ± 0.34 Aa	2.58 ± 0.34 Ba	2.25 ± 0.29 Ba	4.05 ± 0.26 Ab	2.66 ± 0.43 Ba	1.46 ± 0.22 Cb
AP (mg kg^−1^)	6.78 ± 0.50 Aa	4.03 ± 0.71 Ba	2.92 ± 0.39 Ba	5.84 ± 0.39 Aa	5.04 ± 0.62 Aa	3.51 ± 0.27 Ba
Soil C:N	9.07 ± 0.57 Ab	10.53 ± 1.37 Ab	11.38 ± 1.04 Aa	20.41 ± 2.73 Aa	18.56 ± 1.69 Aa	16.58 ± 2.38 Aa
Soil C:P	13.63 ± 1.33 Aa	9.71 ± 0.52 Ba	7.64 ± 0.77 Bb	13.85 ± 0.81 Aa	11.53 ± 0.85 ABa	11.06 ± 1.13 Ba
Soil N:P	1.50 ± 0.08 Aa	1.03 ± 0.09 Ba	0.71 ± 0.08 Ca	0.74 ± 0.06 Ab	0.66 ± 0.06 Ab	0.72 ± 0.08 Aa
MBC (mg kg^−1^)	253.67 ± 13.60 Aa	166.61 ± 12.29 Ba	87.84 ± 9.50 Ca	142.05 ± 9.09 Ab	99.09 ± 7.61 Bb	70.32 ± 6.39 Ca
MBN (mg kg^−1^)	111.27 ± 5.18 Aa	129.78 ± 7.67 Aa	74.10 ± 14.20 Ba	108.78 ± 8.87 Aa	102.56 ± 4.21 Ab	80.82 ± 5.52 Ba
MBP (mg kg^−1^)	8.79 ± 0.90 Aa	7.13 ± 1.13 ABa	5.39 ± 0.96 Ba	7.93 ± 0.71 Aa	8.01 ± 1.10 Aa	4.44 ± 0.66 Ba
MBC:MBN	2.30 ± 0.13 Aa	1.34 ± 0.14 Ba	1.44 ± 0.18 Ba	1.38 ± 0.14 Ab	0.97 ± 0.08 Bb	0.91 ± 0.11 Bb
MBC:MBP	31.86 ± 4.04 Aa	31.77 ± 6.30 Aa	18.41 ± 2.02 Aa	20.72 ± 4.19 Aa	15.18 ± 2.50 Ab	17.98 ± 2.15 Aa
MBN:MBP	13.68 ± 1.28 Ba	24.32 ± 4.46 Aa	14.57 ± 2.47 Ba	15.32 ± 2.43 Aa	16.04 ± 2.54 Aa	22.81 ± 4.19 Aa

Note: Different uppercase letters indicate the significance among the three salinity levels, and different lowercase letters indicate the significance between the rhizosphere and the bulk soils. The data shown are the mean ± standard errors. SOC, organic carbon soil; TN, total nitrogen; TP, total phosphorus; DOC, dissolved organic carbon; NH_4_^+^-N, ammonium nitrogen; NO_3_^−^-N, nitrate nitrogen; AP, available phosphorus; MBC, microbial biomass carbon; MBN, microbial biomass nitrogen; MBP, microbial biomass phosphorus.

**Table 2:** Sample points information.

Sample Site	Longitude	Latitude	Altitude (m)	Main Companion Species
1	117.68°	38.01°	9	*Aeluropus sinensis*, *Artemisia capillaris*, *S. salsa*, *C. chinense*, *Atriplex centralasiatica*, *Setaria viridis*, *Lactuca tatarica*
2	117.95°	37.89°	12	*A. capillaris*, *L. tatarica*, *Plantago asiatica*, *Polygonum aviculare*
3	118.03°	37.99°	8	*S. salsa*, *C. chinense*, *Limonium bicolor*, *A. sinensis*, *A. capillaris*, *I. cylindrical*
4	118.03°	37.74°	14	*I. cylindrical*, *P. asiatica*, *Taraxacum mongolicum*, *Aster altaicus*, *Digitaria sanguinalis*
5	118.06°	38.17°	2	*C. chinense*, *S. salsa*, *P. australis*
6	118.29°	37.40°	9	*C. chinense*, *A. capillaris*, *L. tatarica*
7	118.30°	37.43°	9	*I. cylindrical*, *L. tatarica*, *P. aviculare*
8	118.31°	37.45°	21	*C. chinense*, *L. tatarica*, *P. aviculare*, *Calamagrostis pseudophragmites*
9	118.33°	37.52°	15	*L. tatarica*, *S. salsa*, *P. australis*
10	118.33°	37.50°	10	*C. chinense*, *A. sinensis*, *A. capillaris*, *I. cylindrical*, *L. tatarica*, *P. asiatica*
11	118.43°	37.50°	9	*S. salsa*, *P. australis*
12	118.55°	37.70°	10	*A. altaicus*, *C. chinense*, *I. cylindrical*
13	118.66°	37.33°	7	*L. bicolor*, *S. viridis*
14	118.68°	38.10°	7	*S. salsa*, *P. australis*
15	118.69°	37.40°	7	*S. salsa*, *P. australis*
16	118.70°	38.07°	7	*C. chinense*, *S. salsa*, *P. australis*
17	118.71°	37.51°	9	*C. chinense*, *A. capillaris*, *I. cylindrical*, *L. tatarica*, *S. salsa*, *P. australis*
18	118.73°	37.65°	15	*C. chinense*, *A. sinensis*, *S. salsa*, *P. australis*, *Echinochloa crusgalli*
19	118.75°	38.05°	8	*S. salsa*, *P. australis*
20	118.80°	38.07°	9	*S. salsa*, *P. australis*
21	118.81°	38.13°	10	*S. salsa*, *P. australis*, *C. chinense*, *L. bicolor*, *Takhtajaniantha mongolica*
22	118.83°	37.90°	8	*Cleistogenes serotina*, *S. viridis*, *Conyza canadensis*, *Viola philippica*
23	118.86°	38.04°	7	*C. chinense*, *L. tatarica*, *S. salsa*, *P. australis*
24	118.97°	37.77°	9	*C. chinense*, *A. sinensis*, *A. capillaris*, *I. cylindrical*,
25	118.98°	37.73°	10	*A. sinensis*, *A. capillaris*, *S. salsa*, *P. australis*
26	119.01°	37.85°	6	*A. sinensis*, *S. salsa*, *P. australis*
27	119.05°	37.85°	10	*S. salsa*, *P. australis*
28	119.11°	37.00°	8	*T. mongolicum*, *Metaplexis japonica*, *Humulus scandens*, *Echinochloa crus-galli*
29	119.17°	37.77°	10	*I. cylindrical*, *S. salsa*, *P. australis*, *C. pseudophragmites*
30	119.24°	37.19°	1	*S. viridis*, *A. capillaris*, *H. scandens*
31	119.37°	37.09°	4	*S. salsa*, *P. australis*
32	119.39°	37.00°	6	*S. salsa*, *P. australis*

## Data Availability

The original contributions presented in the study are included in the article. Further inquiries can be directed to the corresponding author.
